# Structure of the Teeth and Extraction Thereof

**Published:** 1848-07

**Authors:** Eri Locke


### STRUCTURE OF THE TEETH AND EXTRACTION THEREOF.

BY ERI LOCKE, D. D. S.

Teeth are small bones fixed in the alveola upper and under jaw. In early infancy nature designs us for the softest aliment, so that the gums alone are then sufficient for the purpose of manducation. But as we advance in life and require different food, (but not animal food,) she wisely provides us with Teeth. These are the hardest and whitest of our bones, (yet require most care,) and at full maturity we have sixteen in each jaw, making in all thirty-two. We often find this number varied from in different subjects. But is seldom more than thirty-two or less than twenty-eight

Each tooth may be divided in two parts: its body or that part which appears above the gums, and its fangs or root which is fixed into the socket of the alveola. There is a marked and distinct boundary between these two. Just at the edge of the gum there is a small circular depression, and by some is called the neck of the tooth.

Anatomists vary in dividing the teeth. Some divide them in but two classes, some three and some four. I prefer making four for I perceive a very marked difference between the incisors and canine or eye teeth, and between the biscuspis and molars.

As every intelligent and observing Dentist is observant of, or acquainted with these varieties of teeth at sight, I will omit a description of each as a separate tooth, and speak of them as a whole.

Every tooth is composed of its cortex (bark) or enamel, and its internal or bony substance. The vitreous part of the tooth is a very hard and compact substance of a white color and very peculiar to the teeth. It is found only upon the body of the teeth covering the outside of the bony or internal substance. When broken it appears striatid, (for this fact I am particularly indebted to my friend Dr. J. F. Potter, who has, during his course in Ohio College Dental Surgery, exhibited specimens, aided with a microscope of power 100, as also several other facts which to me were not clear, but now are perfectly so. The small sum which he received from my hands is not compensation.) And all the stria are denoted from the circumference to the centre of the tooth. The enamel is thickest on the grinding surface, and on the cutting edges of the teeth becoming gradually thinner as it approaches the neck, where it terminates insensibly. Some have said it was vascular, but it is certain no injection will reach this substance, neither can it receive any tinge from madder.

As the enamel surrounds a bony structure, and that without a doubt is vascular and capable of receiving coloring matter, it is quite probable its color has been reflected to the enamel, that substance being translucent has given it an injected appearance. The bony part resembles other bones in its structure, but is much harder than the most compact part of bones in general. Some have said this bone could not be vascular. Such may be the case, but I am impressed with a contrary view. The facts adduced by Mr. Hunter are capable of a different explanation from that which he has given them, and when other facts are added relative to the same subject, it will appear that this bony part of a tooth has a circulation through its substance, and even sympathies, although from the hardness of its structure more unable to demonstrate its vessels.

The facts which may be adduced are, 1st. We find that a tooth recently drawn and transplanted into another socket, becomes as firmly fixed after a certain time, and preserves the same color as the rest of the set; whereas, a tooth that has been long drawn before being transplanted will never become firmly fixed. Mr. Hunter indeed is aware of this objection, and refers the success of the transplantation in the first instance to the living principle possessed by the tooth, and which he thinks may exist independent of a circulation. 2dly. The swellings of the fangs of a tooth, which in many instances are known to be the effects of disease, and which are analagous to the swelling of other bones, are a clear proof of a similarity of structure, especially as we find them invested with a peritoneum. 3dly. It is a curious fact, though as yet not generally known, that in cases of phthis pulmonalis, the teeth become of a milky whiteness, and in some degree transparent. Does not this prove them to have absorbents?

I here before leaving this subject make an assertion which I ask to be proven. If the tooth does not possess absorbents, or vascularity, why does it decay from an injury, and why do anatomists admit of its being organic? It must be inorganic if it possesses no vascularity or absorbents.

We might say that the teeth were nothing but a superabundance of matter without organism, thrown out by nature, and that it has no affinity with the organization of man. Yet, who would not scorn such an idea? In the progress of decay we can observe its radiations in different directions, as it follows up the minute channels which convey to the tooth its life, and by the same channels its death. Each tooth has an inner cavity which, beginning by a small opening at the point of the fang, becomes larger and terminates in the body of the tooth. This cavity is supplied with blood vessels and nerves which pass through the small hole at the extremity of the fang or root. In aged people this hole frequently becomes closed, and the tooth insensible.

The progress in the formation of the teeth, from the time of foetal conception to maturity, I shall leave for others, not that I deem it a subject unworthy of notice in this place, but because I find other subjects which demand my attention.

*Their Extraction.* There is hardly in the practice of a Dentist an operation which he is more frequently called upon to perform than that of extracting, and how few there are who can boast of perfection; and in consequence, this is a subject which makes a demand upon the profession for new developements.

I was agreeably impressed with the reading of an article on the subject by Dr. Cone, and before I close this chapter I shall take occasion to quote from his article, and hope I shall be indulged.

In the extraction of teeth it is very desirable to have a proper quantity of instruments, and especially such as are best adapted to the shape of the superior portion of the teeth.

I am sorry to say that an idea is prevalent among the country practitioners, that if they have a gum lancet, one pair of "hawk-bill forceps," and the turnkey, that is all that is sufficient, providing they have a good "large hand" and *a grip*, they can fetch any thing in the shape of a tooth, that may present in any of their (unfortunate) patients. The learned and intelligent practitioner will have an instrument fitted to almost every variety of teeth which present. He will have these instruments neatly arranged upon a case, table or stand; he will have a chair so arranged as to sit his patient with ease; after having examined the tooth to be extracted and determined the amount of strength necessary for its extraction, he will carefully but deeply pass his lancet around the neck of the tooth, thereby loosening the attachments of the gum; after which apply the forceps carefully, and with a rotary motion of the hand and a pull at the same instant, you will very readily extract the tooth. If the tooth be much decayed it should be grasped as high up under the gum as possible, and no more pressure applied to the handle of the instrument than is necessary to prevent from slipping. Teeth are often unnecessarily broken by not attending to this precaution. A much greater force is required for the extraction of molars, bicuspides, and cuspids than incisors. A much greater amount of force is required for the extraction of the upper molars than of any of the other teeth. Roots of teeth are generally more easily removed than whole teeth. Nevertheless they sometimes are exceedingly difficult of removal, and require instruments different from those used in the extraction of whole teeth—these are the hooks, punch, elevator and screw. every dentist has them somewhat differently constructed, according to his own peculiar fancy or taste. I am not capable of giving any good, better, best, mode of using them—can only say that the necessary tact for the skillful use of these instruments—can only be acquired by a long and observing practice. Dr. Snell recommends an instrument which was in vogue fourteen or fifteen years ago, called the double elevator; the Dr. recommends it very highly for the removal of the dens sapientia of the lower jaw.

I have found in my very limited practice that much depends upon the position of the patient at the time of operating, and in reviewing Dr. Harris' work, I find he mentions the very fact. The operator should stand upon the right side of his patient, (and not before him,) and to insure success must make a careful adjustment of his forceps upon the tooth which is to be removed before applying the force. In judging of the firmness of the teeth, I am led to believe with Dr. Cone, that they vary in their firmness in different persons of different temperaments, and much depends upon the temperaments in forming our judgment as to the amount of force required for the removal of teeth. Dr. Cone says that persons whose temperaments are equally balanced and possess a perfect state of health, physical and mental. In such persons, he says, you will find short crowns, and firmly fixed within the alveola processes. Such teeth are usually crowded in their arrangement in their alveolar arches, which are usually prominent and thin, and not unfrequently from this cause the anterior superior teeth made to strike perpendicularly on the inferior teeth or within the arch of the same. The color of such teeth may be either a light cream or a muddy white, and when the latter exists, their structure is such as to render their crowns very brittle. Teeth of this description are generally found with individuals having thin prominent countenances and energetic features.

